# Bis(2,2′-bipyridine)-1κ^2^ *N*,*N*′;3κ^2^ *N*,*N*′-hexa-μ-methacrylato-1:2κ^6^ *O*:*O*′;2:3κ^6^ *O*:*O*′-(nitrato-2κ^2^ *O*,*O*′)-1,3-dicobalt(II)-2-terbium(III)

**DOI:** 10.1107/S1600536810031053

**Published:** 2010-08-11

**Authors:** Bin Wu, Cheng-Xin Zhao

**Affiliations:** a Department of Chemistry, Zhejiang Sci-Tech University, Hangzhou 310018, People’s Republic of China

## Abstract

In the title trinuclear cobalt–terbium complex, [Co_2_Tb(C_4_H_5_O_2_)_6_(NO_3_)(C_10_H_8_N_2_)_2_], the central Tb^III^ and each of the Co^II^ ions are bridged by three carboxyl­ate groups of the methacrylate anions. The Tb^III^ cation is coordinated by six O atoms from six methacrylate anions and two O atoms from a chelating nitrate anion in a distorted square-anti­prismatic geometry. Each Co^II^ ion is coordinated by three O atoms from three methyl­acrylate anions and two N atoms of a 2,2′-bypiridine ligand in a distorted square-pyramidal geometry. In the crystal structure, π–π stacking between the pyridine rings [centroid–centroid distances = 3.682 (8) and 3.760 (8) Å] is observed and weak inter­molecular C—H⋯O hydrogen bonding is also present.

## Related literature

For the crystal structures of analogous complexes, see: Wu & Guo (2004 ▶); Zhu *et al.* (2005 ▶); Wu (2008 ▶); Wu & Hou (2010 ▶). For details of the preparation of Tb*L*_3_·H_2_O (H*L* = CH_2_C(CH_3_)COOH), see: Lu *et al.* (1995 ▶).
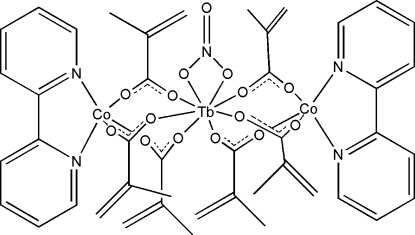


## Experimental

### 

#### Crystal data

[Co_2_Tb(C_4_H_5_O_2_)_6_(NO_3_)(C_10_H_8_N_2_)_2_]
*M* *_r_* = 1161.64
Triclinic, 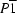

*a* = 11.3717 (6) Å
*b* = 13.4396 (5) Å
*c* = 16.3572 (8) Å
α = 103.912 (2)°
β = 99.950 (2)°
γ = 99.845 (3)°
*V* = 2330.26 (19) Å^3^
*Z* = 2
Mo *K*α radiation
μ = 2.28 mm^−1^
*T* = 293 K
0.32 × 0.31 × 0.15 mm

#### Data collection

Rigaku R-AXIS RAPID diffractometer
Absorption correction: multi-scan (*ABSCOR*; Higashi, 1995 ▶) *T*_min_ = 0.495, *T*_max_ = 0.709
13172 measured reflections
8051 independent reflections
7272 reflections with *I* > 2σ(*I*)
*R*_int_ = 0.031

#### Refinement

*R*[*F* ^2^ > 2σ(*F* ^2^)] = 0.025
*wR*(*F* ^2^) = 0.060
*S* = 1.03
8051 reflections
610 parameters
H-atom parameters constrained
Δρ_max_ = 0.45 e Å^−3^
Δρ_min_ = −0.87 e Å^−3^

### 

Data collection: *RAPID-AUTO* (Rigaku, 1998 ▶); cell refinement: *RAPID-AUTO*; data reduction: *CrystalStructure* (Rigaku/MSC, 2002 ▶); program(s) used to solve structure: *SHELXS97* (Sheldrick, 2008 ▶); program(s) used to refine structure: *SHELXL97* (Sheldrick, 2008 ▶); molecular graphics: *ORTEP-3* (Farrugia, 1997 ▶); software used to prepare material for publication: *SHELXL97* and *PLATON* (Spek, 2009 ▶).

## Supplementary Material

Crystal structure: contains datablocks global, I. DOI: 10.1107/S1600536810031053/xu5010sup1.cif

Structure factors: contains datablocks I. DOI: 10.1107/S1600536810031053/xu5010Isup2.hkl

Additional supplementary materials:  crystallographic information; 3D view; checkCIF report

## Figures and Tables

**Table 1:** Hydrogen-bond geometry (Å, °)

*D*—H⋯*A*	*D*—H	H⋯*A*	*D*⋯*A*	*D*—H⋯*A*
C8—H8⋯O9^i^	0.93	2.46	3.299 (4)	150
C9—H9⋯O7^i^	0.93	2.54	3.291 (4)	138
C38—H38⋯O11^ii^	0.93	2.58	3.457 (4)	157
C42—H42⋯O9^iii^	0.93	2.42	3.259 (5)	150
C43—H43⋯O8^iii^	0.93	2.57	3.316 (4)	138

Symmetry codes: (i) 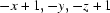; (ii) 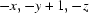; (iii) 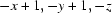.

**Fig. 1.**
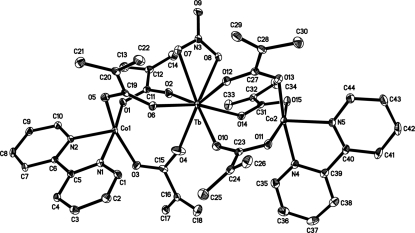
View of the molecule showing the atom-labelling scheme. Displacement ellipsoids are drawn at the 30% probability level. All H atoms have been omitted for clarity.

## supplementary crystallographic information

### Comment

The study of heterometallic complexes containing d-transition metal and lanthanide(III) cations connected by bridging ligands is being actively pursued because of their relevance in solid-state technology and as models for magnetic studies. As a contribution to a structural study of heterometallic complexes containing d-transition metal and rare-earth(III) cations (Wu & Guo, 2004; Zhu *et al.*, 2005; Wu, 2008), herewith we report the synthesis and crystal structure of the title compound, (I).

The crystal structure of the title Co—Tb—Co trinuclear complex is similar to the known crystal structures of the Zn—Ce—Zn, Zn—Nd—Zn, Co—Gd—Co and Co—Ce—Co complexes (Wu & Guo, 2004; Zhu *et al.*, 2005; Wu, 2008; Wu & Hou, 2010). The Tb^III^ center is coordinated by six O atoms from six methacrylato ligands and two O atoms from nitrate anion in a distorted square-antiprismatic geometry. Each Co^II^ ion is coordinated by three O atoms from three methacrylato ligands and two N atoms from 2,2'-bypiridine ligand in a distorted pyramidal geometry. The Tb^III^ and each of two Co^II^ ions are bridged by three bidentate methacrylato ligands. Two Tb···Co separations are almost equal. The separations of Tb···Co1 and Tb···Co2 are 3.937 (1) and 3.822 (1) Å, respectively.

In the crystal structure, π-π interactions between the aromatic rings [centroid-centroid distances of 3.682 (8) and 3.760 (8) Å, respectively] link molecules into chains propagated in direction [01–1]. The aromatic stacking interactions are responsible for the supramolecular assemblies. Weak intermolecular C—H···O hydrogen bonds stabilize further the crystal packing (Table 1).

### Experimental

TbL_3_.H_2_O (864 mg, 2.0 mmol; HL = CH_2_C(CH_3_)COOH) and Co(NO_3_)_2_.6H_2_O (435 mg, 1.5 mmol) were dissolved in 15 ml water, and the pH adjusted to 4.0 using HL. An ethanol solution (3 ml) of 2,2'-bipyridine (234 mg, 1.5 mmol) was added into the above solution with stirring. After filtration, the filtrate was allowed to stand at room temperature and single crystals suitable for X-ray work were obtained after two weeks.

### Refinement

H atoms were placed in idealized locations with C–H distances 0.93 - 0.96 Å and refined as riding with U_iso_(H) = 1.2U_eq_(C) or 1.5U_eq_(C).

### Crystal data

**Table:**

[Co_2_Tb(C_4_H_5_O_2_)_6_(NO_3_)(C_10_H_8_N_2_)_2_]	*Z* = 2
*M**_r_* = 1161.64	*F*(000) = 1168
Triclinic, *P*1	*D*_x_ = 1.656 Mg m^−^^3^
Hall symbol: -P 1	Mo *K*α radiation, λ = 0.71069 Å
*a* = 11.3717 (6) Å	Cell parameters from 10681 reflections
*b* = 13.4396 (5) Å	θ = 2.0–27.5°
*c* = 16.3572 (8) Å	µ = 2.28 mm^−^^1^
α = 103.912 (2)°	*T* = 293 K
β = 99.950 (2)°	Block, brown
γ = 99.845 (3)°	0.32 × 0.31 × 0.15 mm
*V* = 2330.26 (19) Å^3^	

### Data collection

**Table:**

Rigaku R-AXIS RAPID diffractometer	8051 independent reflections
Radiation source: fine-focus sealed tube	7272 reflections with *I* > 2σ(*I*)
graphite	*R*_int_ = 0.031
Detector resolution: 10.00 pixels mm^-1^	θ_max_ = 25.1°, θ_min_ = 1.3°
ω scans	*h* = −13→13
Absorption correction: multi-scan (*ABSCOR*; Higashi, 1995)	*k* = −16→16
*T*_min_ = 0.495, *T*_max_ = 0.709	*l* = −19→19
13172 measured reflections	

### Refinement

**Table:**

Refinement on *F*^2^	Primary atom site location: structure-invariant direct methods
Least-squares matrix: full	Secondary atom site location: difference Fourier map
*R*[*F*^2^ > 2σ(*F*^2^)] = 0.025	Hydrogen site location: inferred from neighbouring sites
*wR*(*F*^2^) = 0.060	H-atom parameters constrained
*S* = 1.03	*w* = 1/[σ^2^(*F*_o_^2^) + (0.0316*P*)^2^ + 2.6217*P*] where *P* = (*F*_o_^2^ + 2*F*_c_^2^)/3
8051 reflections	(Δ/σ)_max_ = 0.001
610 parameters	Δρ_max_ = 0.45 e Å^−^^3^
0 restraints	Δρ_min_ = −0.87 e Å^−^^3^

### Special details

**Table:**

Geometry. All e.s.d.'s (except the e.s.d. in the dihedral angle between two l.s. planes) are estimated using the full covariance matrix. The cell e.s.d.'s are taken into account individually in the estimation of e.s.d.'s in distances, angles and torsion angles; correlations between e.s.d.'s in cell parameters are only used when they are defined by crystal symmetry. An approximate (isotropic) treatment of cell e.s.d.'s is used for estimating e.s.d.'s involving l.s. planes.
Refinement. Refinement of *F*^2^ against ALL reflections. The weighted *R*-factor *wR* and goodness of fit *S* are based on *F*^2^, conventional *R*-factors *R* are based on *F*, with *F* set to zero for negative *F*^2^. The threshold expression of *F*^2^ > σ(*F*^2^) is used only for calculating *R*-factors(gt) *etc*. and is not relevant to the choice of reflections for refinement. *R*-factors based on *F*^2^ are statistically about twice as large as those based on *F*, and *R*- factors based on ALL data will be even larger.

### Fractional atomic coordinates and isotropic or equivalent isotropic displacement parameters (Å^2^)

**Table:**

	*x*	*y*	*z*	*U*_iso_*/*U*_eq_	
Tb	0.349851 (12)	0.235738 (10)	0.240894 (8)	0.01582 (5)	
Co1	0.28569 (3)	0.09556 (3)	0.41926 (2)	0.01591 (9)	
Co2	0.28401 (3)	0.42261 (3)	0.10445 (2)	0.01763 (9)	
O1	0.45584 (18)	0.17936 (16)	0.47967 (13)	0.0221 (4)	
O2	0.47217 (19)	0.26143 (17)	0.37769 (13)	0.0268 (5)	
O3	0.19771 (19)	0.21432 (16)	0.45824 (14)	0.0253 (5)	
O4	0.2203 (2)	0.2614 (3)	0.33945 (16)	0.0486 (7)	
O5	0.33886 (19)	−0.01681 (16)	0.33430 (13)	0.0237 (5)	
O6	0.2821 (2)	0.06055 (17)	0.23263 (16)	0.0308 (5)	
O7	0.5251 (2)	0.14495 (17)	0.22325 (14)	0.0272 (5)	
O8	0.56465 (19)	0.31214 (17)	0.23419 (14)	0.0253 (5)	
O9	0.7096 (2)	0.2241 (2)	0.22484 (17)	0.0405 (6)	
O10	0.15112 (19)	0.22751 (18)	0.16450 (14)	0.0270 (5)	
O11	0.13244 (19)	0.30122 (16)	0.05503 (14)	0.0243 (5)	
O12	0.35412 (19)	0.19272 (17)	0.09420 (13)	0.0255 (5)	
O13	0.38403 (19)	0.33206 (16)	0.04124 (13)	0.0235 (4)	
O14	0.36667 (18)	0.41464 (16)	0.24671 (13)	0.0229 (4)	
O15	0.43760 (19)	0.53596 (16)	0.18666 (13)	0.0253 (5)	
N1	0.1041 (2)	−0.00293 (19)	0.36608 (15)	0.0189 (5)	
N2	0.2646 (2)	0.01087 (18)	0.50865 (15)	0.0173 (5)	
N3	0.6023 (2)	0.2265 (2)	0.22695 (17)	0.0263 (6)	
N4	0.1622 (2)	0.5164 (2)	0.14918 (16)	0.0225 (5)	
N5	0.2614 (2)	0.50765 (19)	0.01371 (16)	0.0208 (5)	
C1	0.0287 (3)	−0.0066 (2)	0.29172 (19)	0.0235 (6)	
H1	0.0560	0.0359	0.2585	0.028*	
C2	−0.0880 (3)	−0.0713 (3)	0.2625 (2)	0.0263 (7)	
H2	−0.1375	−0.0727	0.2104	0.032*	
C3	−0.1289 (3)	−0.1337 (2)	0.31281 (19)	0.0234 (6)	
H3	−0.2066	−0.1778	0.2950	0.028*	
C4	−0.0520 (3)	−0.1294 (2)	0.39046 (19)	0.0215 (6)	
H4	−0.0783	−0.1698	0.4255	0.026*	
C5	0.0650 (3)	−0.0639 (2)	0.41511 (18)	0.0183 (6)	
C6	0.1558 (3)	−0.0565 (2)	0.49490 (18)	0.0180 (6)	
C7	0.1328 (3)	−0.1151 (2)	0.55283 (19)	0.0221 (6)	
H7	0.0568	−0.1598	0.5438	0.027*	
C8	0.2248 (3)	−0.1057 (2)	0.6238 (2)	0.0262 (7)	
H8	0.2109	−0.1443	0.6627	0.031*	
C9	0.3378 (3)	−0.0386 (2)	0.6366 (2)	0.0248 (7)	
H9	0.4009	−0.0320	0.6834	0.030*	
C10	0.3538 (3)	0.0187 (2)	0.57728 (19)	0.0226 (6)	
H10	0.4291	0.0640	0.5853	0.027*	
C11	0.5147 (3)	0.2422 (2)	0.44716 (18)	0.0184 (6)	
C12	0.6439 (3)	0.2969 (2)	0.4958 (2)	0.0235 (6)	
C13	0.6891 (3)	0.2726 (3)	0.5778 (2)	0.0353 (8)	
H13A	0.7746	0.3050	0.5992	0.053*	
H13B	0.6776	0.1978	0.5676	0.053*	
H13C	0.6443	0.2992	0.6196	0.053*	
C14	0.7116 (3)	0.3622 (3)	0.4627 (3)	0.0372 (8)	
H14A	0.7919	0.3953	0.4915	0.045*	
H14B	0.6783	0.3745	0.4109	0.045*	
C15	0.1775 (3)	0.2680 (2)	0.40533 (18)	0.0217 (6)	
C16	0.0906 (3)	0.3387 (2)	0.4226 (2)	0.0232 (6)	
C17	0.0866 (3)	0.3841 (2)	0.5151 (2)	0.0293 (7)	
H17A	0.0281	0.4279	0.5176	0.044*	
H17B	0.1661	0.4254	0.5467	0.044*	
H17C	0.0631	0.3282	0.5401	0.044*	
C18	0.0152 (4)	0.3511 (3)	0.3553 (3)	0.0415 (9)	
H18A	0.0174	0.3163	0.2993	0.050*	
H18B	−0.0394	0.3946	0.3644	0.050*	
C19	0.3180 (2)	−0.0150 (2)	0.2567 (2)	0.0206 (6)	
C20	0.3335 (3)	−0.1085 (2)	0.19035 (19)	0.0209 (6)	
C21	0.3480 (3)	−0.2036 (2)	0.2209 (2)	0.0299 (7)	
H21A	0.3576	−0.2581	0.1740	0.045*	
H21B	0.2767	−0.2282	0.2406	0.045*	
H21C	0.4190	−0.1853	0.2674	0.045*	
C22	0.3347 (3)	−0.1025 (3)	0.1107 (2)	0.0348 (8)	
H22A	0.3449	−0.1595	0.0695	0.042*	
H22B	0.3252	−0.0411	0.0960	0.042*	
C23	0.0991 (3)	0.2310 (2)	0.09099 (19)	0.0219 (6)	
C24	−0.0130 (3)	0.1450 (2)	0.0413 (2)	0.0246 (7)	
C25	−0.0923 (3)	0.1017 (3)	0.0924 (3)	0.0394 (9)	
H25A	−0.1544	0.0431	0.0550	0.059*	
H25B	−0.1304	0.1549	0.1200	0.059*	
H25C	−0.0440	0.0788	0.1355	0.059*	
C26	−0.0316 (3)	0.1097 (3)	−0.0457 (2)	0.0336 (8)	
H26A	−0.0978	0.0549	−0.0767	0.040*	
H26B	0.0217	0.1402	−0.0745	0.040*	
C27	0.3746 (3)	0.2350 (2)	0.03536 (19)	0.0215 (6)	
C28	0.3872 (3)	0.1660 (2)	−0.04942 (19)	0.0227 (6)	
C29	0.3929 (3)	0.0663 (3)	−0.0578 (2)	0.0327 (8)	
H29A	0.4009	0.0247	−0.1100	0.039*	
H29B	0.3888	0.0381	−0.0115	0.039*	
C30	0.3930 (3)	0.2180 (3)	−0.1208 (2)	0.0328 (8)	
H30A	0.3975	0.1674	−0.1721	0.049*	
H30B	0.4643	0.2745	−0.1038	0.049*	
H30C	0.3210	0.2452	−0.1321	0.049*	
C31	0.4425 (3)	0.5002 (2)	0.25101 (19)	0.0206 (6)	
C32	0.5337 (3)	0.5618 (2)	0.33372 (19)	0.0216 (6)	
C33	0.5287 (3)	0.5358 (3)	0.4066 (2)	0.0303 (7)	
H33A	0.5833	0.5756	0.4579	0.036*	
H33B	0.4707	0.4778	0.4064	0.036*	
C34	0.6245 (3)	0.6542 (3)	0.3283 (2)	0.0351 (8)	
H34A	0.6748	0.6901	0.3847	0.053*	
H34B	0.5814	0.7014	0.3067	0.053*	
H34C	0.6751	0.6299	0.2899	0.053*	
C35	0.1151 (3)	0.5158 (3)	0.2190 (2)	0.0286 (7)	
H35	0.1427	0.4767	0.2551	0.034*	
C36	0.0271 (3)	0.5709 (3)	0.2390 (2)	0.0364 (8)	
H36	−0.0031	0.5698	0.2882	0.044*	
C37	−0.0150 (3)	0.6276 (3)	0.1847 (2)	0.0353 (8)	
H37	−0.0749	0.6647	0.1967	0.042*	
C38	0.0321 (3)	0.6291 (2)	0.1125 (2)	0.0282 (7)	
H38	0.0045	0.6670	0.0753	0.034*	
C39	0.1215 (3)	0.5727 (2)	0.09632 (19)	0.0221 (6)	
C40	0.1818 (3)	0.5716 (2)	0.02266 (19)	0.0215 (6)	
C41	0.1620 (3)	0.6338 (3)	−0.0332 (2)	0.0308 (7)	
H41	0.1064	0.6769	−0.0268	0.037*	
C42	0.2259 (3)	0.6308 (3)	−0.0981 (2)	0.0362 (8)	
H42	0.2129	0.6711	−0.1363	0.043*	
C43	0.3096 (3)	0.5670 (3)	−0.1056 (2)	0.0328 (8)	
H43	0.3549	0.5651	−0.1479	0.039*	
C44	0.3244 (3)	0.5060 (3)	−0.0484 (2)	0.0267 (7)	
H44	0.3799	0.4627	−0.0535	0.032*	

### Atomic displacement parameters (Å^2^)

**Table:**

	*U*^11^	*U*^22^	*U*^33^	*U*^12^	*U*^13^	*U*^23^
Tb	0.01728 (8)	0.01693 (8)	0.01758 (8)	0.00519 (5)	0.00563 (5)	0.01065 (5)
Co1	0.01583 (19)	0.01565 (19)	0.01852 (19)	0.00300 (15)	0.00508 (15)	0.00846 (15)
Co2	0.0185 (2)	0.0171 (2)	0.01853 (19)	0.00386 (15)	0.00212 (16)	0.00884 (15)
O1	0.0193 (10)	0.0230 (11)	0.0249 (11)	0.0003 (9)	0.0041 (9)	0.0119 (9)
O2	0.0259 (11)	0.0333 (13)	0.0215 (11)	0.0032 (10)	0.0023 (9)	0.0127 (9)
O3	0.0244 (11)	0.0215 (11)	0.0310 (12)	0.0075 (9)	0.0049 (9)	0.0082 (9)
O4	0.0281 (13)	0.088 (2)	0.0312 (14)	0.0155 (14)	0.0114 (11)	0.0144 (14)
O5	0.0238 (11)	0.0247 (11)	0.0223 (11)	0.0069 (9)	0.0054 (9)	0.0050 (9)
O6	0.0237 (11)	0.0229 (12)	0.0546 (15)	0.0070 (9)	0.0135 (11)	0.0227 (11)
O7	0.0278 (12)	0.0296 (12)	0.0331 (12)	0.0115 (10)	0.0109 (10)	0.0187 (10)
O8	0.0252 (11)	0.0279 (12)	0.0307 (12)	0.0072 (9)	0.0108 (9)	0.0186 (9)
O9	0.0241 (12)	0.0699 (19)	0.0515 (15)	0.0244 (12)	0.0213 (12)	0.0427 (14)
O10	0.0198 (11)	0.0374 (13)	0.0260 (11)	0.0060 (9)	0.0033 (9)	0.0144 (10)
O11	0.0226 (11)	0.0178 (11)	0.0316 (11)	0.0018 (9)	0.0011 (9)	0.0108 (9)
O12	0.0299 (12)	0.0320 (12)	0.0210 (11)	0.0125 (10)	0.0091 (9)	0.0128 (9)
O13	0.0245 (11)	0.0234 (11)	0.0260 (11)	0.0081 (9)	0.0072 (9)	0.0099 (9)
O14	0.0210 (10)	0.0177 (11)	0.0317 (11)	0.0051 (9)	0.0063 (9)	0.0093 (9)
O15	0.0273 (11)	0.0231 (11)	0.0255 (11)	0.0027 (9)	0.0005 (9)	0.0128 (9)
N1	0.0193 (12)	0.0189 (13)	0.0202 (12)	0.0039 (10)	0.0057 (10)	0.0079 (10)
N2	0.0173 (12)	0.0188 (12)	0.0186 (12)	0.0048 (10)	0.0057 (10)	0.0091 (10)
N3	0.0224 (14)	0.0377 (16)	0.0293 (14)	0.0118 (12)	0.0092 (11)	0.0227 (12)
N4	0.0224 (13)	0.0229 (13)	0.0218 (13)	0.0050 (11)	0.0020 (11)	0.0077 (10)
N5	0.0189 (12)	0.0223 (13)	0.0228 (13)	0.0031 (10)	0.0029 (10)	0.0116 (10)
C1	0.0220 (15)	0.0286 (17)	0.0229 (15)	0.0039 (13)	0.0067 (13)	0.0127 (13)
C2	0.0219 (16)	0.0348 (18)	0.0227 (15)	0.0051 (13)	0.0025 (13)	0.0113 (13)
C3	0.0195 (15)	0.0198 (15)	0.0269 (16)	0.0002 (12)	0.0048 (13)	0.0021 (12)
C4	0.0238 (15)	0.0168 (15)	0.0265 (16)	0.0045 (12)	0.0088 (13)	0.0085 (12)
C5	0.0220 (15)	0.0133 (14)	0.0214 (14)	0.0054 (11)	0.0075 (12)	0.0050 (11)
C6	0.0200 (15)	0.0163 (14)	0.0220 (14)	0.0085 (12)	0.0092 (12)	0.0071 (11)
C7	0.0249 (16)	0.0177 (15)	0.0281 (16)	0.0038 (12)	0.0101 (13)	0.0121 (12)
C8	0.0337 (18)	0.0255 (17)	0.0280 (16)	0.0108 (14)	0.0094 (14)	0.0184 (13)
C9	0.0275 (16)	0.0280 (17)	0.0232 (15)	0.0124 (13)	0.0040 (13)	0.0123 (13)
C10	0.0197 (15)	0.0238 (16)	0.0274 (16)	0.0062 (12)	0.0047 (13)	0.0124 (13)
C11	0.0195 (14)	0.0166 (14)	0.0191 (14)	0.0061 (11)	0.0055 (12)	0.0026 (11)
C12	0.0187 (15)	0.0205 (15)	0.0276 (16)	0.0026 (12)	0.0027 (13)	0.0029 (12)
C13	0.0255 (17)	0.047 (2)	0.0315 (18)	0.0077 (15)	−0.0025 (15)	0.0140 (16)
C14	0.0226 (17)	0.036 (2)	0.053 (2)	−0.0016 (15)	0.0057 (16)	0.0198 (17)
C15	0.0170 (14)	0.0263 (16)	0.0187 (15)	0.0002 (12)	0.0057 (12)	0.0028 (12)
C16	0.0232 (15)	0.0193 (15)	0.0312 (16)	0.0041 (12)	0.0099 (13)	0.0125 (13)
C17	0.0368 (18)	0.0188 (16)	0.0389 (18)	0.0107 (14)	0.0166 (15)	0.0113 (14)
C18	0.047 (2)	0.041 (2)	0.045 (2)	0.0197 (18)	0.0093 (18)	0.0233 (18)
C19	0.0125 (13)	0.0190 (15)	0.0313 (17)	−0.0016 (11)	0.0078 (12)	0.0105 (12)
C20	0.0158 (14)	0.0231 (16)	0.0223 (15)	0.0007 (12)	0.0048 (12)	0.0058 (12)
C21	0.0326 (18)	0.0189 (16)	0.0344 (18)	0.0053 (13)	0.0050 (15)	0.0028 (13)
C22	0.0280 (18)	0.047 (2)	0.0315 (18)	0.0074 (16)	0.0087 (15)	0.0140 (16)
C23	0.0184 (15)	0.0221 (16)	0.0283 (16)	0.0085 (12)	0.0061 (13)	0.0093 (13)
C24	0.0200 (15)	0.0199 (16)	0.0340 (17)	0.0054 (12)	0.0006 (13)	0.0111 (13)
C25	0.0281 (18)	0.040 (2)	0.050 (2)	0.0012 (16)	0.0056 (17)	0.0205 (18)
C26	0.0332 (18)	0.0247 (17)	0.0382 (19)	0.0045 (14)	−0.0039 (15)	0.0096 (14)
C27	0.0143 (14)	0.0299 (17)	0.0223 (15)	0.0062 (12)	0.0021 (12)	0.0115 (13)
C28	0.0182 (15)	0.0315 (17)	0.0195 (15)	0.0042 (13)	0.0064 (12)	0.0087 (13)
C29	0.0376 (19)	0.0330 (19)	0.0327 (18)	0.0079 (15)	0.0194 (16)	0.0105 (15)
C30	0.0382 (19)	0.040 (2)	0.0220 (16)	0.0088 (16)	0.0088 (15)	0.0095 (14)
C31	0.0179 (14)	0.0176 (15)	0.0280 (16)	0.0079 (12)	0.0040 (12)	0.0077 (12)
C32	0.0224 (15)	0.0210 (15)	0.0230 (15)	0.0082 (12)	0.0038 (12)	0.0073 (12)
C33	0.042 (2)	0.0243 (17)	0.0219 (16)	0.0064 (14)	0.0029 (14)	0.0044 (13)
C34	0.0314 (18)	0.034 (2)	0.0337 (18)	−0.0055 (15)	−0.0004 (15)	0.0124 (15)
C35	0.0292 (17)	0.0303 (18)	0.0254 (16)	0.0033 (14)	0.0048 (14)	0.0094 (13)
C36	0.0314 (19)	0.045 (2)	0.0309 (18)	0.0080 (16)	0.0091 (15)	0.0054 (16)
C37	0.0272 (18)	0.0343 (19)	0.040 (2)	0.0126 (15)	0.0056 (15)	−0.0004 (15)
C38	0.0268 (17)	0.0203 (16)	0.0325 (17)	0.0050 (13)	−0.0004 (14)	0.0032 (13)
C39	0.0179 (15)	0.0167 (15)	0.0259 (15)	0.0006 (12)	−0.0041 (12)	0.0037 (12)
C40	0.0225 (15)	0.0148 (14)	0.0251 (15)	0.0008 (12)	−0.0002 (12)	0.0079 (12)
C41	0.0335 (18)	0.0233 (17)	0.0366 (18)	0.0075 (14)	−0.0008 (15)	0.0156 (14)
C42	0.046 (2)	0.0308 (19)	0.0366 (19)	0.0034 (16)	0.0041 (16)	0.0246 (16)
C43	0.0317 (18)	0.038 (2)	0.0320 (18)	0.0003 (15)	0.0083 (15)	0.0210 (15)
C44	0.0255 (16)	0.0283 (17)	0.0282 (16)	0.0046 (13)	0.0057 (14)	0.0126 (13)

### Geometric parameters (Å, °)

**Table:**

Tb—O6	2.312 (2)	C13—H13A	0.9600
Tb—O2	2.334 (2)	C13—H13B	0.9600
Tb—O12	2.340 (2)	C13—H13C	0.9600
Tb—O10	2.356 (2)	C14—H14A	0.9300
Tb—O14	2.357 (2)	C14—H14B	0.9300
Tb—O4	2.369 (2)	C15—C16	1.499 (4)
Tb—O8	2.514 (2)	C16—C18	1.337 (5)
Tb—O7	2.530 (2)	C16—C17	1.501 (4)
Co1—O1	2.012 (2)	C17—H17A	0.9600
Co1—O5	2.042 (2)	C17—H17B	0.9600
Co1—O3	2.057 (2)	C17—H17C	0.9600
Co1—N2	2.078 (2)	C18—H18A	0.9300
Co1—N1	2.160 (2)	C18—H18B	0.9300
Co2—O13	2.029 (2)	C19—C20	1.509 (4)
Co2—O11	2.051 (2)	C20—C22	1.327 (4)
Co2—N5	2.088 (2)	C20—C21	1.503 (4)
Co2—O15	2.115 (2)	C21—H21A	0.9600
Co2—N4	2.133 (3)	C21—H21B	0.9600
Co2—O14	2.389 (2)	C21—H21C	0.9600
O1—C11	1.260 (3)	C22—H22A	0.9300
O2—C11	1.260 (3)	C22—H22B	0.9300
O3—C15	1.269 (4)	C23—C24	1.518 (4)
O4—C15	1.248 (4)	C24—C26	1.354 (5)
O5—C19	1.257 (4)	C24—C25	1.473 (5)
O6—C19	1.277 (4)	C25—H25A	0.9600
O7—N3	1.263 (3)	C25—H25B	0.9600
O8—N3	1.282 (3)	C25—H25C	0.9600
O9—N3	1.232 (3)	C26—H26A	0.9300
O10—C23	1.260 (4)	C26—H26B	0.9300
O11—C23	1.266 (4)	C27—C28	1.516 (4)
O12—C27	1.265 (4)	C28—C29	1.328 (5)
O13—C27	1.269 (4)	C28—C30	1.503 (4)
O14—C31	1.293 (4)	C29—H29A	0.9300
O15—C31	1.254 (4)	C29—H29B	0.9300
N1—C1	1.346 (4)	C30—H30A	0.9600
N1—C5	1.352 (4)	C30—H30B	0.9600
N2—C10	1.347 (4)	C30—H30C	0.9600
N2—C6	1.350 (4)	C31—C32	1.508 (4)
N4—C35	1.343 (4)	C32—C33	1.328 (4)
N4—C39	1.352 (4)	C32—C34	1.503 (4)
N5—C44	1.338 (4)	C33—H33A	0.9300
N5—C40	1.353 (4)	C33—H33B	0.9300
C1—C2	1.390 (4)	C34—H34A	0.9600
C1—H1	0.9300	C34—H34B	0.9600
C2—C3	1.389 (4)	C34—H34C	0.9600
C2—H2	0.9300	C35—C36	1.381 (5)
C3—C4	1.395 (4)	C35—H35	0.9300
C3—H3	0.9300	C36—C37	1.379 (5)
C4—C5	1.396 (4)	C36—H36	0.9300
C4—H4	0.9300	C37—C38	1.382 (5)
C5—C6	1.489 (4)	C37—H37	0.9300
C6—C7	1.401 (4)	C38—C39	1.393 (4)
C7—C8	1.386 (4)	C38—H38	0.9300
C7—H7	0.9300	C39—C40	1.484 (4)
C8—C9	1.389 (5)	C40—C41	1.394 (4)
C8—H8	0.9300	C41—C42	1.383 (5)
C9—C10	1.392 (4)	C41—H41	0.9300
C9—H9	0.9300	C42—C43	1.387 (5)
C10—H10	0.9300	C42—H42	0.9300
C11—C12	1.509 (4)	C43—C44	1.391 (4)
C12—C14	1.341 (5)	C43—H43	0.9300
C12—C13	1.483 (4)	C44—H44	0.9300
			
O6—Tb—O2	89.72 (8)	C14—C12—C13	123.8 (3)
O6—Tb—O12	91.10 (8)	C14—C12—C11	119.2 (3)
O2—Tb—O12	141.89 (7)	C13—C12—C11	116.9 (3)
O6—Tb—O10	86.68 (8)	C12—C13—H13A	109.5
O2—Tb—O10	144.54 (7)	C12—C13—H13B	109.5
O12—Tb—O10	73.50 (7)	H13A—C13—H13B	109.5
O6—Tb—O14	165.64 (7)	C12—C13—H13C	109.5
O2—Tb—O14	97.02 (7)	H13A—C13—H13C	109.5
O12—Tb—O14	91.36 (7)	H13B—C13—H13C	109.5
O10—Tb—O14	80.47 (7)	C12—C14—H14A	120.0
O6—Tb—O4	83.07 (10)	C12—C14—H14B	120.0
O2—Tb—O4	73.78 (8)	H14A—C14—H14B	120.0
O12—Tb—O4	144.05 (8)	O4—C15—O3	124.3 (3)
O10—Tb—O4	70.76 (8)	O4—C15—C16	119.4 (3)
O14—Tb—O4	86.65 (9)	O3—C15—C16	116.2 (3)
O6—Tb—O8	122.83 (7)	C18—C16—C15	118.5 (3)
O2—Tb—O8	73.60 (7)	C18—C16—C17	123.8 (3)
O12—Tb—O8	74.10 (7)	C15—C16—C17	117.5 (3)
O10—Tb—O8	136.02 (7)	C16—C17—H17A	109.5
O14—Tb—O8	71.39 (7)	C16—C17—H17B	109.5
O4—Tb—O8	137.65 (9)	H17A—C17—H17B	109.5
O6—Tb—O7	71.84 (7)	C16—C17—H17C	109.5
O2—Tb—O7	71.68 (7)	H17A—C17—H17C	109.5
O12—Tb—O7	72.45 (7)	H17B—C17—H17C	109.5
O10—Tb—O7	138.97 (7)	C16—C18—H18A	120.0
O14—Tb—O7	122.32 (7)	C16—C18—H18B	120.0
O4—Tb—O7	136.89 (8)	H18A—C18—H18B	120.0
O8—Tb—O7	51.00 (7)	O5—C19—O6	122.9 (3)
O1—Co1—O5	95.93 (8)	O5—C19—C20	117.3 (3)
O1—Co1—O3	96.07 (8)	O6—C19—C20	119.8 (3)
O5—Co1—O3	156.98 (8)	C22—C20—C21	124.4 (3)
O1—Co1—N2	96.20 (9)	C22—C20—C19	119.4 (3)
O5—Co1—N2	96.25 (9)	C21—C20—C19	116.2 (2)
O3—Co1—N2	101.89 (9)	C20—C21—H21A	109.5
O1—Co1—N1	173.49 (8)	C20—C21—H21B	109.5
O5—Co1—N1	84.80 (9)	H21A—C21—H21B	109.5
O3—Co1—N1	85.48 (9)	C20—C21—H21C	109.5
N2—Co1—N1	77.30 (9)	H21A—C21—H21C	109.5
O13—Co2—O11	89.61 (9)	H21B—C21—H21C	109.5
O13—Co2—N5	94.45 (9)	C20—C22—H22A	120.0
O11—Co2—N5	100.68 (9)	C20—C22—H22B	120.0
O13—Co2—O15	94.95 (9)	H22A—C22—H22B	120.0
O11—Co2—O15	164.39 (8)	O10—C23—O11	125.1 (3)
N5—Co2—O15	93.85 (9)	O10—C23—C24	118.0 (3)
O13—Co2—N4	170.11 (9)	O11—C23—C24	116.9 (3)
O11—Co2—N4	86.32 (9)	C26—C24—C25	123.9 (3)
N5—Co2—N4	77.49 (10)	C26—C24—C23	119.1 (3)
O15—Co2—N4	91.34 (9)	C25—C24—C23	117.0 (3)
O13—Co2—O14	97.41 (8)	C24—C25—H25A	109.5
O11—Co2—O14	106.35 (8)	C24—C25—H25B	109.5
N5—Co2—O14	150.45 (9)	H25A—C25—H25B	109.5
O15—Co2—O14	58.29 (7)	C24—C25—H25C	109.5
N4—Co2—O14	92.38 (8)	H25A—C25—H25C	109.5
C11—O1—Co1	121.33 (18)	H25B—C25—H25C	109.5
C11—O2—Tb	158.9 (2)	C24—C26—H26A	120.0
C15—O3—Co1	115.23 (19)	C24—C26—H26B	120.0
C15—O4—Tb	162.7 (2)	H26A—C26—H26B	120.0
C19—O5—Co1	116.41 (19)	O12—C27—O13	124.7 (3)
C19—O6—Tb	141.31 (19)	O12—C27—C28	118.6 (3)
N3—O7—Tb	95.80 (17)	O13—C27—C28	116.6 (3)
N3—O8—Tb	96.05 (16)	C29—C28—C30	123.5 (3)
C23—O10—Tb	138.55 (19)	C29—C28—C27	120.7 (3)
C23—O11—Co2	125.38 (19)	C30—C28—C27	115.8 (3)
C27—O12—Tb	141.3 (2)	C28—C29—H29A	120.0
C27—O13—Co2	124.09 (18)	C28—C29—H29B	120.0
C31—O14—Tb	143.88 (18)	H29A—C29—H29B	120.0
C31—O14—Co2	84.09 (17)	C28—C30—H30A	109.5
Tb—O14—Co2	107.30 (8)	C28—C30—H30B	109.5
C31—O15—Co2	97.56 (18)	H30A—C30—H30B	109.5
C1—N1—C5	119.0 (2)	C28—C30—H30C	109.5
C1—N1—Co1	126.30 (19)	H30A—C30—H30C	109.5
C5—N1—Co1	114.68 (19)	H30B—C30—H30C	109.5
C10—N2—C6	119.5 (2)	O15—C31—O14	120.0 (3)
C10—N2—Co1	123.40 (19)	O15—C31—C32	118.6 (3)
C6—N2—Co1	117.13 (18)	O14—C31—C32	121.3 (3)
O9—N3—O7	121.5 (3)	C33—C32—C34	123.5 (3)
O9—N3—O8	121.4 (3)	C33—C32—C31	120.3 (3)
O7—N3—O8	117.1 (2)	C34—C32—C31	116.2 (3)
C35—N4—C39	118.8 (3)	C32—C33—H33A	120.0
C35—N4—Co2	125.8 (2)	C32—C33—H33B	120.0
C39—N4—Co2	115.0 (2)	H33A—C33—H33B	120.0
C44—N5—C40	119.4 (3)	C32—C34—H34A	109.5
C44—N5—Co2	124.1 (2)	C32—C34—H34B	109.5
C40—N5—Co2	116.35 (19)	H34A—C34—H34B	109.5
N1—C1—C2	122.8 (3)	C32—C34—H34C	109.5
N1—C1—H1	118.6	H34A—C34—H34C	109.5
C2—C1—H1	118.6	H34B—C34—H34C	109.5
C3—C2—C1	118.4 (3)	N4—C35—C36	122.4 (3)
C3—C2—H2	120.8	N4—C35—H35	118.8
C1—C2—H2	120.8	C36—C35—H35	118.8
C2—C3—C4	119.1 (3)	C37—C36—C35	118.7 (3)
C2—C3—H3	120.4	C37—C36—H36	120.6
C4—C3—H3	120.4	C35—C36—H36	120.6
C3—C4—C5	119.3 (3)	C36—C37—C38	119.7 (3)
C3—C4—H4	120.3	C36—C37—H37	120.1
C5—C4—H4	120.3	C38—C37—H37	120.1
N1—C5—C4	121.3 (3)	C37—C38—C39	118.8 (3)
N1—C5—C6	115.1 (2)	C37—C38—H38	120.6
C4—C5—C6	123.7 (3)	C39—C38—H38	120.6
N2—C6—C7	120.9 (3)	N4—C39—C38	121.5 (3)
N2—C6—C5	115.7 (2)	N4—C39—C40	115.0 (3)
C7—C6—C5	123.3 (3)	C38—C39—C40	123.5 (3)
C8—C7—C6	119.1 (3)	N5—C40—C41	121.1 (3)
C8—C7—H7	120.4	N5—C40—C39	115.6 (2)
C6—C7—H7	120.4	C41—C40—C39	123.3 (3)
C7—C8—C9	119.9 (3)	C42—C41—C40	119.4 (3)
C7—C8—H8	120.1	C42—C41—H41	120.3
C9—C8—H8	120.1	C40—C41—H41	120.3
C10—C9—C8	118.0 (3)	C43—C42—C41	119.3 (3)
C10—C9—H9	121.0	C43—C42—H42	120.4
C8—C9—H9	121.0	C41—C42—H42	120.4
N2—C10—C9	122.5 (3)	C42—C43—C44	118.6 (3)
N2—C10—H10	118.7	C42—C43—H43	120.7
C9—C10—H10	118.7	C44—C43—H43	120.7
O2—C11—O1	124.1 (3)	N5—C44—C43	122.2 (3)
O2—C11—C12	119.0 (3)	N5—C44—H44	118.9
O1—C11—C12	116.9 (3)	C43—C44—H44	118.9

### Hydrogen-bond geometry (Å, °)

**Table:**

*D*—H···*A*	*D*—H	H···*A*	*D*···*A*	*D*—H···*A*
C8—H8···O9^i^	0.93	2.46	3.299 (4)	150
C9—H9···O7^i^	0.93	2.54	3.291 (4)	138
C38—H38···O11^ii^	0.93	2.58	3.457 (4)	157
C42—H42···O9^iii^	0.93	2.42	3.259 (5)	150
C43—H43···O8^iii^	0.93	2.57	3.316 (4)	138

Symmetry codes: (i) −*x*+1, −*y*, −*z*+1; (ii) −*x*, −*y*+1, −*z*; (iii) −*x*+1, −*y*+1, −*z*.
